# Innovative Lipid Nanoparticles Co-Delivering Hydroxychloroquine and siRNA for Enhanced Rheumatoid Arthritis Therapy

**DOI:** 10.3390/pharmaceutics17010045

**Published:** 2025-01-01

**Authors:** Yanru Feng, Xintong Pan, Ziqian Li, Yue Li, Ya’nan Sun, Shaokun Yang, Chaoxing He, Yunjie Dang, Lu Huang, Bai Xiang

**Affiliations:** 1 Department of Pharmaceutics, School of Pharmaceutical Sciences, Hebei Medical University, Shijiazhuang 050017, China; fengyru@stu.hebmu.edu.cn (Y.F.); 22033100158@stu.hebmu.edu.cn (X.P.); lzq15733699460@163.com (Z.L.); yueli@stu.hebmu.edu.cn (Y.L.); sunyn@hebmu.edu.cn (Y.S.); yangshaokun@hebmu.edu.cn (S.Y.); chaoxinghe@hebmu.edu.cn (C.H.); dangyunjie@hebmu.edu.cn (Y.D.); 2 Hebei Key Laboratory of Innovative Drug Research and Evaluation, Hebei Medical University, Shijiazhuang 050017, China

**Keywords:** lipid nanoparticle, co-delivery, hydroxychloroquine, si*TNF-α*, rheumatoid arthritis

## Abstract

**Background:** Rheumatoid arthritis (RA) is a debilitating autoimmune disorder characterized by chronic inflammation and joint damage. Despite advancements in treatment, complete remission remains elusive. **Methods:** In this study, we introduce a novel lipid nanoparticle formulation co-delivering hydroxychloroquine (HCQ) and siRNA targeting TNF-α (si*TNF-α*) using microfluidic technology, marking the first use of such a combination for RA therapy. **Results:** In LPS-stimulated RAW 264.7 cells, the nanoparticles effectively reduced inflammatory markers. When administered via an intra-articular injection in a rat model, they significantly decreased joint inflammation and demonstrated good biological safety. **Conclusions:** This pioneering approach highlights the potential of lipid nanoparticles as a dual-delivery platform for enhanced RA treatment through targeted intra-articular administration.

## 1. Introduction

Rheumatoid arthritis (RA) is an autoimmune disease of unknown etiology dominated by inflammatory synovitis. RA is characterized by polyarticular, symmetrical, and aggressive joint inflammation, frequently accompanied by the excessive production of pro-inflammatory cytokines, which can lead to long-standing synovitis, joint destruction, disability, decreased quality of life, and other comorbidities [1,2,3,4]. Tumor necrosis factor-α (TNF-α), one of the essential pro-inflammatory cytokines mainly secreted by macrophages, is significantly upregulated in RA, leading to the persistence of inflammation, formation of the inflammatory microenvironment, and severe joint damage [5,6,7]. Based on the critical function of TNF-α in RA, therapies that can inhibit TNF-α hold great potential for RA treatment.

Currently, no permanent treatment is available for RA [8]. The primary goal of the available options is to improve the patient’s quality of life by achieving low disease activity or remission [9]. The therapeutic options include the following: (1) glucocorticoids as prednisolone; (2) conventional synthetic disease-modifying antirheumatic drugs (DMARDs) such as hydroxychloroquine (HCQ), methotrexate, and sulfasalazine; (3) biological targeted DMARDs (bDMARD), including the tumor necrosis factor (TNF)-α inhibitors such as etanercept, infliximab, and adalimumab; interleukin (IL)-6 receptor inhibitors such as tocilizumab and sarilumab; and immunomodulators, including abatacept and rituximab; Janus kinase inhibitors, such as baricitinib and tofacitinib, etc. [10,11]. Once known only as an antimalarial drug, HCQ is among the few small-molecular drugs used for RA therapy that have been reported to relieve RA symptoms via several different pharmacological mechanisms: inhibiting lysosomal activity and autophagy, reducing the function of antigen-presenting cells, inhibiting the production of pro-inflammatory cytokines (such as TNF-α), and deactivating signal pathways [12,13]. However, these drugs also have some side effects in the treatment of RA, such as hepatotoxicity, cytopenia, transaminase elevation, gastrointestinal effects, lymphopenia, neutropenia, elevated cholesterol, infections, etc. [13,14]. Moreover, there is still a vast number of patients who do not respond positively to the existing therapeutic strategies. Therefore, there is an urgent need for new, safer, and more effective treatments to halt the progression of the disease and reverse the pathological process.

Lipid nanoparticles (LNPs) are the most widely studied non-viral vectors for siRNA delivery due to the commercialization of Onpattro^®^ (patisiran), a siRNA-LNP, for the treatment of polyneuropathy [15,16,17]. LNPs typically consist of four components, i.e., ionizable lipid, phospholipid, cholesterol, and PEG lipid, which self-assemble with siRNAs to form LNPs [18,19,20]. LNPs can protect siRNAs from degradation by RNase, promote intracellular delivery and endosomal escape of siRNAs, and have low toxicity and biocompatibility [21,22]. In this study, we used LNPs to encapsulate HCQ and si*TNF-α* simultaneously. In brief, the ethanol phases (MC3, Chol, DSPC, DSPE-PEG_2000_) and acidic aqueous phases (si*TNF-α*, HCQ) are mixed with a microfluidic device at a volume ratio of 1:3 to complete the self-assembly process, after which the acidic buffer solution is replaced with PBS (pH = 7.4) [23]. Under acidic conditions, ionizable lipid MC3 is positively charged and can interact with negatively charged siRNA electrostatically to form LNPs. At the same time, HCQ in an aqueous solution is also encapsulated in LNPs. After the replacement of the buffer solution, LNPs were incubated with HCQ at 60 °C, and the encapsulate efficiency of HCQ was further improved by the transmembrane pH gradient method [24,25,26]. The objective of this study was to investigate the therapeutic effect of LNP-based co-delivery of HCQ and si*TNF-α* on RA. Small interfering RNA (siRNA) can specifically silence gene expression via RNA interference [27,28,29]. Using TNF-α-targeting siRNA (si*TNF-α*) cytokine to suppress the production of the pro-inflammatory TNF-α cytokine selectively is a novel approach for the treatment of RA [30,31,32]. HCQ has anti-inflammatory effects by inhibiting the production of TNF-α, IL-1, IL-6, and IFN-γ [12,33,34]. Therefore, we used the LNP co-delivery of HCQ and si*TNF-α* to treat rats with adjuvant-induced arthritis (AIA).

In this study, we innovatively established an LNP co-delivery system of the small-molecular drug HCQ and the gene drug si*TNF-α* for RA treatment. In vitro, LNP-si*TNF-α*-HCQ incubated with LPS-stimulated RAW 264.7 cells significantly reduced TNF-α in the cell supernatant. In vivo, after treatment with LNP-si*TNF-α*-HCQ, the paw volume, arthritis scores, and plasma TNF-α content of the established arthritis model rats were significantly lower than those of the control group. Simultaneously, blood biochemical analysis and H&E staining results showed that LNP-si*TNF-α*-HCQ did not damage blood cells and reduce liver and kidney functions, which proved that LNP-si*TNF-α*-HCQ was not only practical but also safe. LNPs were used to successfully co-deliver HCQ and si*TNF-α* for the treatment of RA. As safe and effective delivery vectors, LNPs can simultaneously encapsulate nucleic acid and small-molecular drugs, providing a new idea for developing RA drugs in the future.

## 2. Materials and Methods

### 2.1. Materials

(6Z,9Z,28Z,31Z)-Heptatriacont-6,9,28,31-tetraene-19-yl 4-(dimethylamino) butanoate (DLin-MC3-DMA), 1,2-distearyol-sn-glycero-3-phosphorylcholine (DSPC), and *N*-(carbonyl-methoxy polyethylene glycol 2000)-1,2-distearoyl-sn-glycerol-3-phosphoethanolamine (DSPE-PEG_2000_) were purchased from AVT Pharmaceutical Technology Co., Ltd. (Shanghai, China). Cholesterol (Chol) was obtained from Nippon Fine Chemical Co., Ltd. (Tomiya, Japan). si*TNF-α* (sense strand: 5′-GUCUCAGCCUCUUCUCAUUCCUGCU-3′; antisense strand: 5′-AGCAGGAAUGAGAAGAGGCUGAGAC-3′) and si*N.C.* (sense strand: 5′-UUCUCCGAACGUGUCACGUTT-3′; antisense strand: 5′-ACGUGACAC GUUCGGAGAATT-3′) were purchased from Genepharma Co., Ltd. (Suzhou, China). Hydroxychloroquine sulfate (HCQ) and ethanol were purchased from Sigma-Aldrich Trading Co., Ltd. (Shanghai, China). Fetal bovine serum (FBS), Dulbecco’s Modified Eagle Medium (DMEM), phosphate-buffered saline (PBS, pH 7.4), penicillin, streptomycin, and trypsin-EDTA were purchased from Gibco (New York, NY, USA). The CCK-8 assay kit was purchased from APE×BIO (Houston, TX, USA). Mouse TNF alpha ELISA (#BMS607-3) and Quant-iT™ RiboGreen RNA Quantitative Kit were acquired from Thermo Fisher Scientific (Waltham, MA, USA). All the reagents were used without any further purification from commercial sources.

### 2.2. Cells and Animals

The murine macrophage cell line RAW 264.7 was maintained in Dulbecco’s Modified Eagle’s Medium (DMEM) and supplemented with 100 U/mL penicillin, 100 μg/mL streptomycin, and 10% (*v*/*v*) fetal bovine serum (FBS). The cells were grown in a 5% CO_2_–95% atmospheric air incubator at 37 °C. The growth medium was renewed every second day, and the cells were sub-cultured twice a week by detaching them from the culture flask and scraping them.

Healthy male Sprague Dawley rats (body weight: 180 ± 20 g) were obtained from SPF Biotechnology Co., Ltd. (Beijing, China). The rats were housed in pathogen-free controlled climatic conditions with artificial lighting (12 h dark–light cycle). The rats were individually kept and acclimatized for 7 days before the experiment. Clean drinking water and a commercially available standard pellet diet were provided ad libitum. To minimize suffering, the rats were anesthetized with isoflurane or pentobarbital sodium (30 mg/kg). The authors have adhered to the ARRIVE guidelines. All experiments were performed as per the Regulations of the Experimental Animal Administration, issued by the State Committee of Science and Technology of the People’s Republic of China (14 November 1988). Ethical approval to conduct the study was obtained from the Laboratory Animal Ethical and Welfare Committee of Hebei Medical University (approval number: IACUC-Hebmu-2024082).

### 2.3. Preparation of LNPs

The organic phase (ethanol solution) consisted of MC3:Chol:DSPC:DSPE-PEG_2000_ (molar ratio) = 30:28.5:40:1.5, and the concentration of MC3, Chol, DSPC, and DSPE-PEG_2000_ was 80.61, 76.58, 107.47, and 4.03 μmoL/L, respectively. The aqueous phase (citrate buffer solution, pH = 4.0) consisted of HCQ (4.8 μg/mL) and si*N.C.*/si*TNF-α* (200 nM). The organic and aqueous phases were mixed at a flow rate of 1:3 by the microfluidic mixing method to collect the mixture (N/P = 10; total flow rate: 4 mL/min). Tangential flow filtration (TFF) was used to replace and concentrate the mixture simultaneously with PBS. In short, the mixture was pumped into the tangential flow membrane package (10 kD) with a peristaltic pump (the inlet pressure was maintained at 2.5 bar). After the replacement was completed, the formulation was collected and incubated with the HCQ aqueous solution at 60 °C for 15 min, and then replaced and concentrated by TFF again to obtain LNP-si*N.C.*-HCQ or LNP-si*TNF-α*-HCQ.

LNP-si*N.C.* and LNP-si*TNF-α* do not add HCQ during two-phase mixing, and there is no incubation process with HCQ after replacement. ^DIR^LNP-siRNA-HCQ was prepared by adding a DIR probe into the ethanol solution, and the other procedures are the same as above.

### 2.4. Characterization of LNPs

LNP particle size distribution and ζ-potential were determined using a Zetasizer Nano ZS90 from Malvern Instrument (Malvern, Worcestershire, UK) based on dynamic light scattering. Measurements were performed in triplicate at an angle of 90° and a temperature of 25 °C. Samples were diluted (1/10) in 1× PBS for size measurements and diluted (1/200) in 0.1× PBS for ζ-potential measurements.

According to the manufacturer’s instructions, the siRNA encapsulation rate was determined using the Quant-iT™ RiboGreen RNA Quantitative Kit (Waltham, MA, USA). HCQ concentrations were analyzed using a high-performance liquid chromatograph (HPLC). Chromatographic separation was performed on a Kromasil 100-5 C18 chromatographic column (250 mm × 4.6 mm, 5 μm), and the column temperature was maintained at 40 °C. The mobile phase comprised 0.05% trifluoroacetic acid in water and methanol (*v/v* = 50/50). The chromatographic separation was achieved using isocratic elution. The flow rate was set to 1 mL/min and the injection volume was 5.0 μL for all samples. The HCQ concentration was measured at 331 nm using a UV detector. The standard curve of HCQ was plotted for concentrations of 0.1, 1, 10, 50, and 100 μg/mL.

LNP morphology was observed by cryo-electron microscopy. About 4 μL of LNP solution was dropped on the copper mesh, and the excess liquid was absorbed with filter paper to form a thin liquid film of solution. The copper mesh was immediately placed in liquid ethane and stored in liquid nitrogen until imaged. Images were obtained at 57–73 K magnifications using a Talos F200C cryo-transmission electron microscope and a 4 × 4 k CMOS camera (Waltham, MA, USA).

### 2.5. Stability of LNPs

Free si*N.C.*, LNP-si*N.C.*, and LNP-si*N.C.*-HCQ were mixed with FBS at a volume ratio of 1:1 and incubated at 37 °C for different times. Samples were taken at 0, 2, 4, 6, 8 and 24 h. A 1 μL volume of 0.5 M EDTA and 4 μL of 2% Triton were added to the samples and frozen at −80 °C. All samples were subjected to agarose gel electrophoresis and imaged. The LNP-si*TNF-α* and LNP-si*TNF-α*-HCQ’s size and encapsulation efficiency of HCQ and si*TNF-α* were measured for 7 days to evaluate the stability of LNPs.

### 2.6. Cytotoxicity

RAW 264.7 cells were plated in a 96-well plate at 5 × 10^3^ cells/well density in a 100 μL complete medium for 24 h. The culture medium was then discarded, and cells were incubated with various concentrations of LNP-si*N.C.*, LNP-si*TNF-α*, LNP-si*N.C.*-HCQ, and LNP-si*TNF-α*-HCQ (si*N.C.*/si*TNF-α* concentrations of 12.5, 25, 50, 100, and 200 nM corresponding to HCQ concentrations of 0.3, 0.6, 1.2, 2.4, and 4.8 ng/mL). After 24 h, the culture medium was discarded, and 100 µL serum-free medium containing 10 µL CCK-8 was added to each well under the light shield. Incubation continued for 4 h, and the microplate reader (Männedorf, Zurich, Switzerland) detected the absorbance OD value at 450 nm.

### 2.7. Cellular Uptake

The cellular uptake of RAW 264.7 was studied using flow cytometry and confocal microscopy. For flow cytometry, 2 mL of the cell suspension at 3 × 10^5^ cells/well was added to the 6-well plates and incubated at 37 °C for 24 h. The culture medium was then discarded, and cells were stimulated with a 2 mL complete medium containing 100 ng/mL LPS for 24 h. Subsequently, the free si*N.C.*-Cy5, LNP-si*N.C.*-Cy5, and LNP-si*N.C.*-Cy5-HCQ were diluted with 2 mL FBS-free medium and added to the cells and incubated for 2 h and 5 h, respectively, at a final concentration of 200 nM si*N.C.*-Cy5 and 4.8 μg/mL HCQ. After incubation, the cells were washed thrice using pre-cooled PBS containing heparin sodium. Then, the cells were collected, washed, and suspended with PBS twice, and analyzed by flow cytometry (Franklin Lake, New Jersey, USA).

RAW 264.7 cells (3 × 10^5^ cells/well) were incubated in a glass bottom dish for 24 h and stimulated by 100 ng/mL LPS for 24 h. Subsequently, the free si*N.C.*-Cy5, LNP-si*N.C.*-Cy5, and LNP-si*N.C.*-Cy5-HCQ were added to the RAW 264.7 cells at 200 nM for si*N.C.*-Cy5 and 4.8 μg/mL for HCQ per well and incubated for 2 h and 5 h, respectively. Next, 500 μL of pre-cooled PBS was added to the well to wash the cells twice. After the removal of PBS, 1 mL of 4% paraformaldehyde solution was added to the wells, and the plates were incubated for 15 min to fix the cells. Next, 1 mL of PBS containing heparin sodium was added and discarded thrice. Furthermore, 500 μL of DAPI solution was added to the cells, and the plates were incubated for 15 min. The wells were then washed using PBS containing heparin sodium three times. Finally, the anti-fluorescence quencher was dropped on the glass bottom dish. After the air bubbles’ removal, the slide was sealed, and the cellular uptake was observed using a confocal laser scanning microscope (CLSM; Baden-Württemberg, Oberkochen, Germany).

### 2.8. The In Vitro Anti-Inflammatory Effect of LNP-siTNF-α-HCQ

Briefly, RAW 264.7 cells were plated in a 96-well plate at 5 × 10^4^ cells/well density in a 100 μL complete medium for 24 h. The culture medium was then discarded, and the cells were stimulated with 100 μL complete medium containing 100 ng/mL LPS for 4 h to establish a cell inflammation model. Subsequently, the cells were washed twice with PBS and cultured in the FBS-free DMEM and incubated with saline, free si*TNF-α*, Free HCQ, LNP-si*TNF-α*, LNP-si*N.C.*-HCQ, and LNP-si*TNF-α*-HCQ (200 nM of siRNA and 4.8 μg/mL of HCQ) for 4 h. Then, 100 μL of 20% FBS medium was added to each well and incubated for 20 h. After that, the culture media were collected and centrifuged at 3000 rpm for 10 min. The levels of TNF-α of supernatants were measured by ELISA kit according to the manufacturer’s instructions.

### 2.9. Establishment of an AIA Rat Model

A 100 μL volume of Freund’s complete adjuvant was injected into the right hind footpad to induce the adjuvant-induced arthritis (AIA) rat model, and immunity was enhanced on the 7th day. Noticeable swelling was observed in the rats’ right hind paw and ankle 10 days later, indicating the successful establishment of the AIA model.

### 2.10. In Vivo Biodistribution

The AIA model was successfully established on the 12th day for the biodistribution study. Rats were intra-articularly administrated with 100 µL of ^DIR^LNP-si*N.C.*-HCQ (si*N.C.* at a dose of 0.03 mg/kg and HCQ at 0.048 mg/kg) into the right ankle joint. At 4, 8, 24, 32, 48, and 96 h after injection, the images were acquired using the IVIS Spectrum Imaging System at the excitation wavelength of 750 nm and the emission wavelength of 780 nm.

### 2.11. In Vivo Therapeutic Efficacy

Twelve days after Freund’s complete adjuvant injection, AIA rats were randomly divided into seven groups. Each group was intra-articularly administrated with 100 µL saline, free si*TNF-α*, Free HCQ, LNP-si*N.C.*, LNP-si*TNF-α*, LNP-si*N.C.*-HCQ, and LNP-si*TNF-α*-HCQ five times every 2 days (si*N.C.* at a dose of 0.03 mg/kg and HCQ at 0.048 mg/kg). Paw volume, paw inflammation, and weight of rats were recorded every two days. On day 22, each group of rats’ right hind paw was photographed and then sacrificed. Blood samples were collected and counted, and liver and kidney functions and inflammatory factors were measured. The numbers of red blood cells (RBCs), white blood cells (WBCs), platelets (PLT), and lymphocytes (Lymphs), as well as aspartate aminotransferase (AST), alanine aminotransferase (ALT), and alkaline phosphatase (ALP) concentrations representing liver function and creatinine (CREA) representing kidney function, were measured by automatic biochemical analyzer. ELISA kits were used to determine the serum concentration of TNF-α. The rat’s main organs (the heart, liver, spleen, lungs, and kidneys) were dissected and fixed with 4% paraformaldehyde for H&E staining. In addition, the body weight of mice for each group was also recorded.

### 2.12. Statistical Analysis

The results are presented as means ± standard deviation (SD). The data were compared by *t*-test between two groups and one-way analysis of variance (ANOVA) for three or more groups. The GraphPad Prism 8.0 software conducted all statistical analyses. A statistically significant difference threshold was defined as * *p* < 0.05, ** *p* < 0.01, *** *p* < 0.001, and ns as no significance.

## 3. Results and Discussion

### 3.1. Characterization of LNP-siTNF-α and LNP-siTNF-α-HCQ

**Figure 1:**
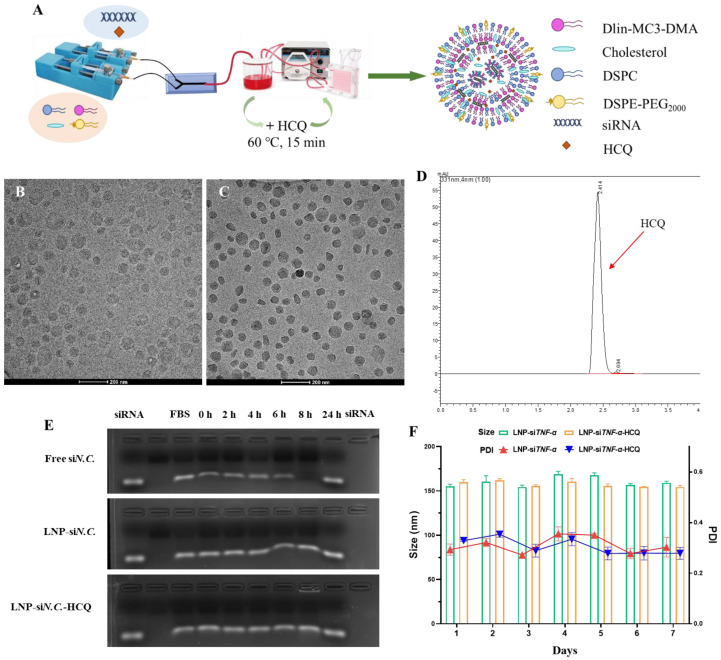
Characterization of LNP-si*TNF-α* and LNP-si*TNF-α*-HCQ. (**A**) Preparation process of LNP-si*TNF-α*-HCQ. Morphology of LNP-si*TNF-α* (**B**) and LNP-si*TNF-α*-HCQ (**C**) characterized by cryo-electron microscopy. (**D**) The high-performance liquid chromatogram of HCQ. (**E**) The serum stability of free si*N.C.*, LNP-si*N.C.*, and LNP-si*N.C.*-HCQ. (**F**) The particle size and PDI stability of LNP-si*TNF-α* and LNP-si*TNF-α*-HCQ.

LNP-si*TNF-α* and LNP-si*TNF-α*-HCQ were prepared through microfluidic formulation, and their final nanoparticles were 155.13 ± 2.51 and 159.83 ± 2.80 nm, respectively, in diameter according to the dynamic light scattering (DLS) analysis. The ζ-potential was −0.65 ± 0.90 and −0.99 ± 0.69 mV for LNP-si*TNF-α* and LNP-si*TNF-α*-HCQ, respectively (Table 1). The cryo-electron microscopy (Cyro-EM) measurement suggested the LNP had a spherical morphology, and LNP-si*TNF-α*-HCQ has a higher electron density (Figure 1A,B). The results showed no difference in particle size between the LNP-si*TNF-α* and LNP-si*TNF-α*-HCQ, indicating that the addition of HCQ did not affect the particle size of LNP. Quant-iT RiboGreen RNA Assay and HPLC further evaluated the encapsulation efficiency (EE%) of HCQ and si*TNF-α*, and the results of EE% are shown in Table 1. They indicated that HCQ and si*TNF-α* were successfully loaded into the LNP. The high-performance liquid chromatogram of HCQ is shown in Figure 1D. The linear equation for HCQ is Y = 12668X − 3226.4, R^2^ = 0.9998 (Y: peak area of HCQ; X: concentration of HCQ).

Subsequently, free si*N.C.*, LNP-si*N.C.*, and LNP-si*N.C.*-HCQ were mixed with fetal bovine serum (FBS, *v*:*v* = 1:1), incubated at 37 °C for 0, 2, 4, 6, 8 and 24 h, and agarose gel electrophoresis (AGE) was performed. AGE results showed that free si*N.C.* was degraded with the prolongation of incubation time and wholly degraded at 24 h. LNP-si*N.C.* and LNP-si*N.C.*-HCQ can protect si*N.C.* from degradation, indicating their specific serum stability (Figure 1E). Similarly, the prepared LNP-si*TNF-α* and LNP-si*TNF-α*-HCQ were placed at 4 °C for 7 days, and appropriate amounts were taken daily to determine their particle size, respectively. As shown in Figure 1F, the particle size of LNP-si*TNF-α* and LNP-si*TNF-α*-HCQ remained stable within 7 days without aggregation. The EE% of LNP-si*TNF-α* and LNP-si*TNF-α*-HCQ on HCQ and si*TNF-α* were measured on day 1 and day 7. The results showed that the EE% of si*TNF-α* (90.36 ± 1.11% and 89.71 ± 1.97%) for LNP-si*TNF-α* and LNP-si*TNF-α*-HCQ and HCQ (17.71 ± 1.75%) for LNP-si*TNF-α*-HCQ on day 7, respectively, showed no significant decrease when compared with the results of day 1 (Table 1).

The above results show that, when LNPs are encapsulated with si*TNF-α* alone or co-encapsulated with HCQ and si*TNF-α*, the LNP particle size and the encapsulation efficiency remain stable within 7 days. This proves that the microfluidic mixing method has successfully prepared LNP-si*TNF-α* and LNP- si*TNF-α*-HCQ. Serra Gürcan et al. developed a single drug delivery system consisting of cationic dexamethasone palmitate nanoparticles associated with si*TNF-α* [35]. To our knowledge, LNPs were used for the first time to simultaneously encapsulate small-molecular drug HCQ and small-nucleic acid drug si*TNF-α*. The HCQ and si*TNF-α* co-delivery system is innovatively established using a microfluidic mixing method. The LNP preparation using the microfluidic mixing method has the advantages of high production efficiency, good repeatability between batches, simple and convenient operation, a high degree of automation, etc.

### 3.2. Cytotoxicity of LNP-siN.C./siTNF-α and LNP-siN.C./siTNF-α-HCQ

**Figure 2:**
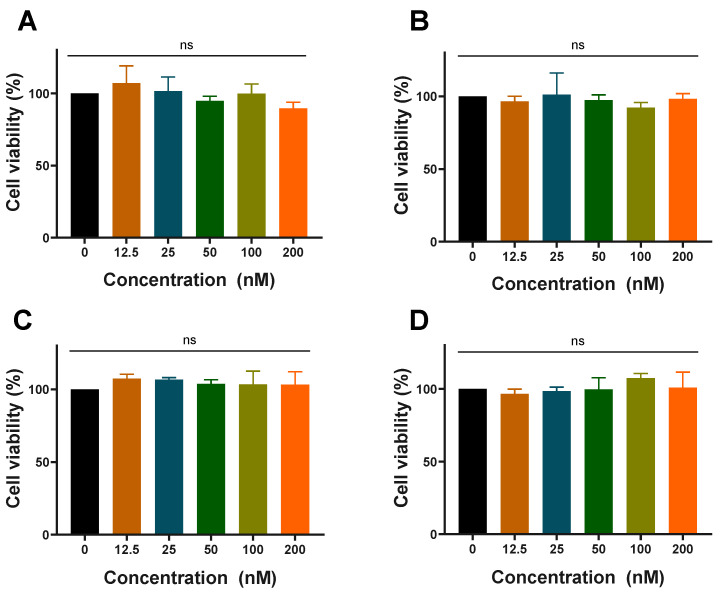
Cytotoxicity in RAW 264.7 cells of LNP-si*N.C.* (**A**), LNP-si*TNF-α* (**B**), LNP-si*N.C.*-HCQ (**C**), and LNP-si*TNF-α*-HCQ (**D**) prepared with different concentrations of siRNA; ns: no significance.

Before evaluating the cellular uptake and anti-inflammatory effect, the cytotoxicity in RAW 264.7 cells was investigated by the CCK-8 assay. RAW 264.7 cells were incubated for 24 h with various concentrations of LNP-si*N.C.*, LNP-si*TNF-α*, LNP-si*N.C.*-HCQ, and LNP-si*TNF-α*-HCQ. At the highest studied concentrations (si*N.C.*/si*TNF-α* at 200 nM and HCQ at 4.8 ng/mL), LNPs showed no significant cytotoxicity toward RAW 264.7 cells, indicating that the LNP-si*N.C.*/si*TNF-α* prepared in this experiment was safe, and the addition of HCQ did not increase its cytotoxicity (Figure 2). LNPs, as non-viral delivery carriers, exhibit cell safety and are used to deliver siRNA and mRNA [36,37]. Therefore, LNPs were selected as the delivery carrier for HCQ and si*TNF-α* in this study.

### 3.3. Cellular Uptake

**Figure 3:**
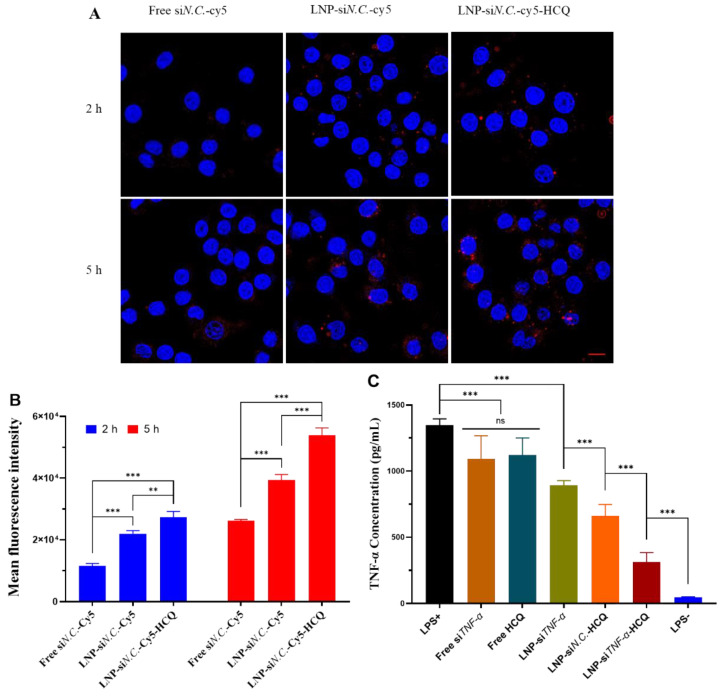
The cellular uptake of free si*N.C.*-Cy5, LNP-si*N.C.*-Cy5, and LNP-si*N.C.*-Cy5-HCQ by RAW 264.7 cells was studied using confocal laser microscopy (**A**) and flow cytometry (**B**). (**C**) The in vitro anti-inflammatory effect of free si*TNF-α*, Free HCQ, LNP-si*TNF-α*, LNP-si*N.C.*-HCQ, and LNP-si*TNF-α*-HCQ on RAW 264.7 cells. Scale bar: 10 μm (**A**); ** *p* < 0.01, *** *p* < 0.001, ns: no significance.

Subsequently, LNPs were prepared using Cy5-labeled si*N.C.* (si*N.C.*-Cy5), and RAW 264.7 cells were selected to assess their cellular uptake. Free si*N.C.*-Cy5, LNP-si*N.C.*-Cy5, and LNP-si*N.C.*-Cy5-HCQ were incubated with RAW 264.7 cells for 2 h and 5 h, respectively, and analyzed by CLSM and flow cytometry. The results of CLSM are shown in Figure 3A, the free si*N.C.*-Cy5 group had almost no red fluorescence (Cy5) after 2 h incubation with RAW 264.7 cells, and only weak fluorescence around individual cells could be seen at 5 h. The red fluorescence of LNP-si*N.C.*-Cy5 and LNP-si*N.C.*-Cy5-HCQ groups was observed in the cytoplasm and near the nucleus after 2h. The fluorescence intensity was stronger after 5 h, indicating that siRNA could be taken up by the RAW 264.7 cells at 2 h. The amount of LNPs taken increased with the extension of incubation time with the cells. Therefore, when LNPs deliver si*TNF-α*, which can silence TNF-α, si*TNF-α* can exert its gene-silencing effect in the cytoplasm after its endosomal escape. Flow cytometry was further performed for quantitative characterization (Figure 3B). RAW 264.7 cells were incubated with free si*N.C.*-Cy5, LNP-si*N.C.*-Cy5, and LNP-si*N.C.*-Cy5-HCQ groups for 2 h. The mean fluorescence intensity (MFI) of the LNP-si*N.C.*-Cy5-HCQ group was significantly higher than that of free si*N.C.*-Cy5 (*p* < 0.001) and LNP-si*N.C.*-Cy5 (*p* < 0.01). Similarly, after incubation for 5 h, the MFI value of the LNP-si*N.C.*-Cy5-HCQ group was significantly higher than that of free si*N.C.*-Cy5 and LNP-si*N.C.*-Cy5, and higher than the MFI of 2 h. These results indicate that LNP-si*N.C.*-Cy5-HCQ has a higher cellular uptake capacity. When LNP-si*N.C.*-Cy5-HCQ is co-cultured with RAW 264.7 cells, LNPs are taken up by the cells and enter into the endosomes, and siRNAs need to escape into the cytoplasm to exert their silencing effect. One of the mechanisms of HCQ is the inhibition of lysosomal activity and autophagy [13,38], which enables it to play an important role in facilitating the endosomal escape of siRNA and its cytoplasmic delivery. Therefore, LNPs that co-encapsulates HCQ and siRNA have a more substantial gene-silencing effect.

### 3.4. Evaluation of the In Vitro Anti-Inflammatory Effect of LNP-siTNF-α-HCQ

To study the anti-inflammatory effect of LNP-si*TNF-α*-HCQ in vitro, an inflammatory model was established by stimulating RAW 264.7 cells with LPS. After various groups were incubated with RAW 264.7 cells, the concentration of TNF-α in the cell medium was determined by the ELISA kit. The results (Figure 3C) showed that RAW 264.7 cells were stimulated by LPS (LPS+), the concentration of TNF-α increased significantly, and the cells non-stimulated by LPS (LPS−) were used as negative control. After treatment with free si*TNF-α* and Free HCQ, the TNF-α concentration decreased when compared with that of the LPS+ group, but there was no difference among the three groups. Similarly, LNP-si*TNF-α*, LNP-si*N.C.*-HCQ, and LNP-si*TNF-α*-HCQ co-cultured with RAW 264.7 cells significantly reduced the amount of TNF-α, and the LNP-si*TNF-α*-HCQ group had the most significant reduction, which was closest to the LPS- group. Compared with LNP-si*TNF-α* and LNP-si*N.C.*-HCQ groups, the content of TNF in the cell medium supernatant was lower in the LNP-si*TNF-α*-HCQ group after incubation with RAW 264.7 cells, indicating that LNPs co-encapsulating HCQ and si*TNF-α* had better anti-inflammatory effect than the single encapsulating HCQ or si*TNF-α* group.

Under physiological conditions, TNF-α has essential functions such as anti-tumor and anti-inflammatory [39]. If TNF-α is released continuously or produced excessively, it will cause the disorder of other cytokines and participate in the progression of the disease. Therefore, the ability of LNP-si*TNF-α*-HCQ to inhibit TNF-α was investigated in vitro. HCQ and si*TNF-α* were co-delivered by LNPs into the cytoplasm, and si*TNF-α* can specifically silence TNF-α, thereby reducing the expression of TNF-α and achieving anti-inflammatory effects [12,30]. Another mechanism of action of HCQ is the inhibition of pro-inflammatory cytokine signaling pathways, ultimately reducing TNF-α production [13,33,38]. Therefore, when LNPs simultaneously encapsulated HCQ and si*TNF-α* and incubated with RAW 264.7 cells, it substantially inhibited TNF-α and achieved the anti-inflammatory purpose.

### 3.5. In Vivo Therapeutic Efficacy

**Figure 4:**
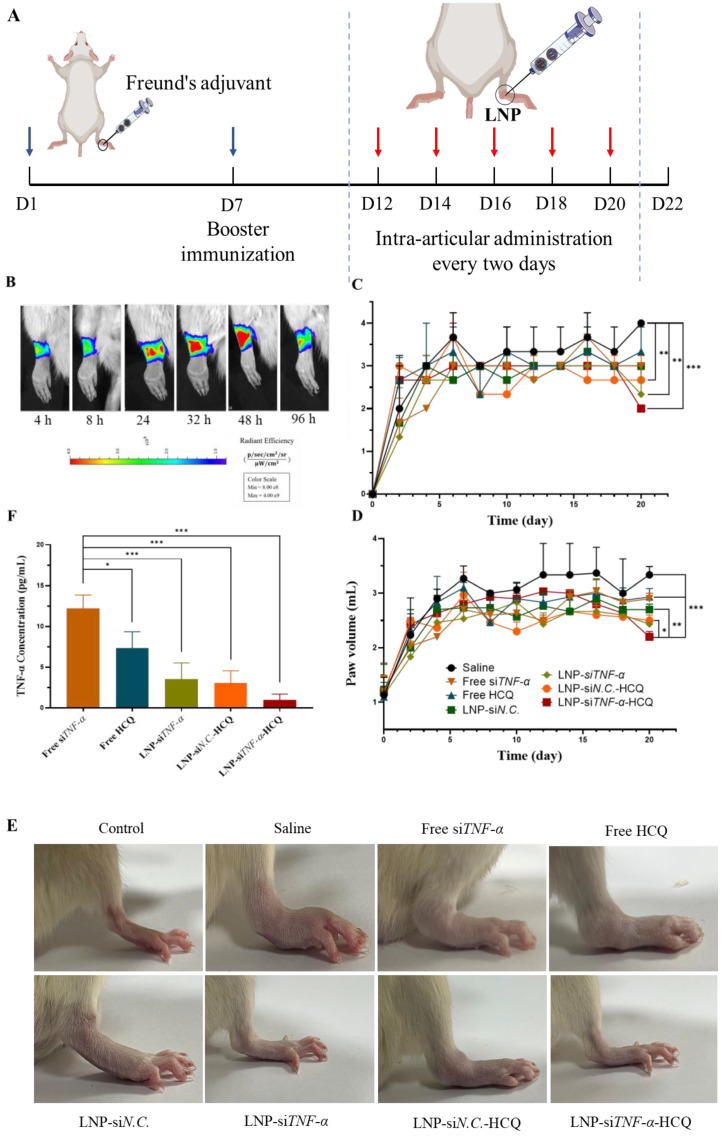
Therapeutic efficacy of LNP-si*TNF-α*-HCQ on adjuvant-induced rheumatoid arthritis rat model. (**A**) Timeline of in vivo study. (**B**) Biodistribution of LNP-si*TNF-α*-HCQ in rheumatoid arthritis rat articular cavity. Arthritis score (**C**), paw volume (**D**), and TNF-α content (**F**) in plasma of AIA rats were assessed in different groups. (**E**) The picture of the rat’s hind paw in different groups before sacrifice. * *p* < 0.05, ** *p* < 0.01, *** *p* < 0.001.

Encouraged by the above results, we hypothesized that LNP-si*N.C.*-Cy5-HCQ would give promising therapeutic efficacy in a model of rheumatoid arthritis. First, the biodistribution of LNP-si*N.C.*-Cy5-HCQ in the ankle joint of rats was studied. IVIS imaging showed that the fluorescence of LNPs could be observed at 4 h after LNP-si*N.C.*-HCQ was injected in the right hind footpad, with the highest fluorescence intensity at 48 h. The above results may be because si*N.C.*-Cy5 was encapsulated in LNPs, the concentration of si*N.C.*-Cy5 was higher, and the fluorescence-quenching phenomenon occurred. When LNP-si*N.C.*-Cy5-HCQ escapes from the endosome into the cytoplasm, si*N.C.*-Cy5 concentration decreases, resulting in fluorescence. Therefore, when studying the therapeutic efficiency of AIA rats, the administration of LNPs every other day was selected (Figure 4A,B).

For in vivo therapeutic efficacy, AIA rats were randomly divided into seven groups. Each group was intra-articularly administrated with saline, free si*TNF-α*, Free HCQ, LNP-si*N.C.*, LNP-si*TNF-α*, LNP-si*N.C.*-HCQ, and LNP-si*TNF-α*-HCQ, and paw volume and paw inflammation were recorded. The arthritis scores were evaluated using a scale of 0–4 to reflect paw inflammation, where 0: normal paw with no erythema or swelling; 1: mild swelling and erythema restricted to the tarsals or ankle joint; 2: mild swelling and erythema extending from the ankle to the tarsus; 3: moderate swelling and erythema extending from the ankle to metatarsal joints; and 4: severe swelling and erythema involving the ankle, feet, and digits, or ankylosis of the limbs [40,41].

The treated groups exhibited varying degrees of alleviation in swelling. The results of joint scores showed that, on day 20 after the establishment of the AIA model (Figure 4C), the average arthritis scores of LNP-si*TNF-α* (2.3), LNP-si*N.C.*-HCQ (2.7), and LNP-si*TNF-α*-HCQ (2.0) groups were significantly lower than those of the saline group (4.0). After five administrations, paw volume measurements also showed a trend consistent with arthritis scores. The LNP-si*TNF-α*-HCQ group significantly reduced paw volume, suggesting that LNP co-delivering HCQ and si*TNF-α* had a more effective therapeutic effect on AIA rats than when si*TNF-α* or HCQ were delivered alone (Figure 4D). At the end of treatment, the hind paws of rats in different groups were photographed, which indicated that the joints of rats treated with LNP-si*TNF-α*-HCQ significantly alleviated the swelling of the paw (Figure 4E).

Cytokines are crucial in regulating the body’s immune and inflammatory responses and are significant factors in synovial inflammation development. Therefore, the serum levels of the pro-inflammatory cytokine TNF-α were measured after the rats were sacrificed. As shown in Figure 4F, the serum TNF-α level in the LNP-si*TNF-α*-HCQ group was significantly lower than that in the free si*TNF-α* group (*p* < 0.001). Overexpressed TNF-α in the joint synovial fluid induce lymphocytes so that they aggregate in the inflammatory joint area, stimulating the release of many pro-inflammatory cytokines. This study was the first to establish the co-delivery system of HCQ and si*TNF-α* using LNPs as carriers. HCQ can inhibit the pro-inflammatory cytokine signaling pathway, and directly or indirectly inhibit the secretion of a variety of pro-inflammatory cytokines, such as TNF-α, which has clinical significance in reducing disease activity and symptoms [37]. A TNF-α-targeting siRNA (si*TNF-α*) is ideal for inhibiting TNF-α production and rescuing RA-damaged joints [31]. Both HCQ and si*TNF-α* can inhibit the production of TNF-α at the inflammatory site of AIA model rats, thereby reducing the inflammation and swelling of rat paws. In this study, LNPs that co-delivered HCQ and si*TNF-α* inhibited the production of pro-inflammatory cytokine TNF-α, and thus had a therapeutic effect on the AIA rat model.

### 3.6. Safety Evaluation

**Figure 5:**
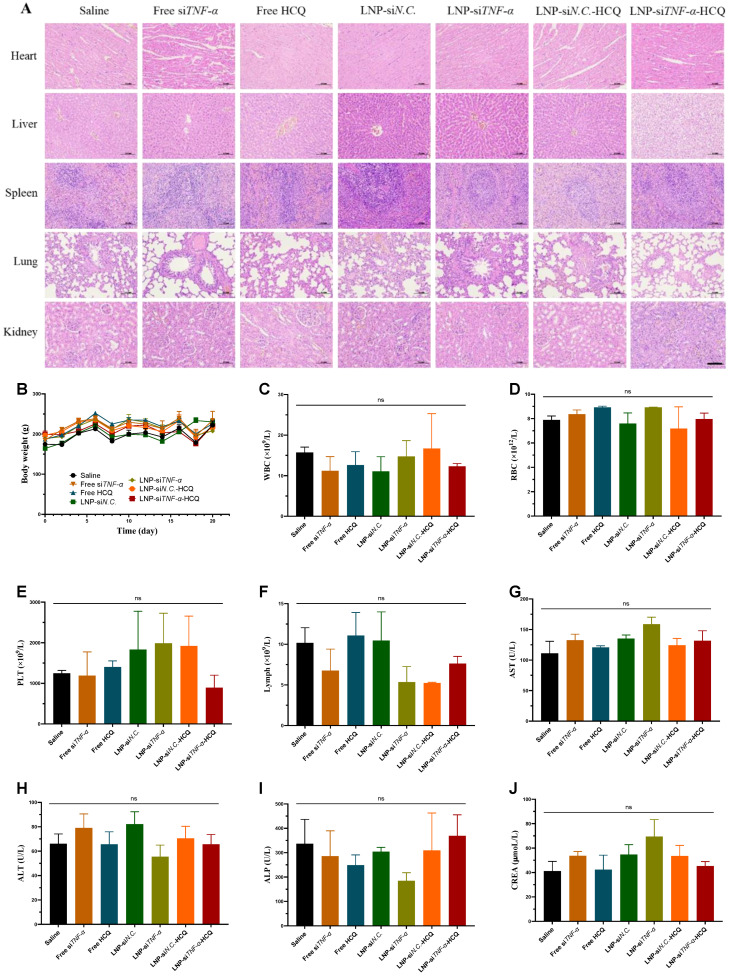
Safety evaluation of AIA rats treated with different administration groups. (**A**) H&E staining of various organs. (**B**) The body weight changes in rats. (**C**–**F**) The number of WBCs, RBCs, PLTs, and Lymphs in the blood after rats were sacrificed. (**G**–**J**) The concentration of AST, ALT, ALP, and CREA in the blood. Scale bar: 100 μm; ns: no significance.

Major organs, including the heart, liver, lung, spleen, and kidneys, were harvested and subjected to H&E staining to determine tissue histopathology. As illustrated in the H&E images (Figure 5A), the rats treated with LNP-si*TNF-α*-HCQ exhibited no noticeable pathological abnormalities or inflammatory lesions in the major organs. The body weight of the rats in each group remained stable without a significant decrease (Figure 5B). Furthermore, whole blood was extracted from the rats at the end of the treatment period and subjected to routine blood tests. As illustrated in Figure 5C–F, the WBC, RBC, PLT, and Lymph numbers remained within the respective normal range. Moreover, serum biochemistry analyses were conducted to assess liver and kidney function parameters. No significant differences were observed among the groups (Figure 5H–J), indicating the absence of acute liver or kidney injury. The results encouraged us to conclude that LNP-si*TNF-α*-HCQ possesses good in vivo biocompatibility.

## 4. Conclusions

In this research, we achieved a breakthrough by successfully devising and comprehensively characterizing a novel lipid nanoparticle system. This system is designed to co-deliver hydroxychloroquine and TNF-α-targeting siRNA to treat rheumatoid arthritis. A meticulously planned synthesis process was employed to create a lipid nanoparticle system capable of simultaneously encapsulating hydroxychloroquine, a commonly utilized small-molecular drug in rheumatoid arthritis treatment, and siRNA directed against TNF-α. Utilizing a suite of cutting-edge techniques and experiments, we conducted an in-depth characterization of this nanoparticle system.

Our experimental results indicated that the newly developed lipid nanoparticle platform exhibits outstanding therapeutic benefits. In contrast to the traditional single-treatment modalities for rheumatoid arthritis, it demonstrated a significantly enhanced capacity to inhibit both the symptoms and the progression of the disease. The combination of hydroxychloroquine and siRNA within the nanoparticles appeared to induce a synergistic effect. Moreover, the nanoparticles exhibited enhanced targeting precision towards the affected tissues, guaranteeing the precise delivery of the drugs to the desired sites, thereby leading to an improved overall treatment efficacy. This elevated therapeutic potential holds great promise, suggesting that this innovative approach could offer a more effective treatment option for patients suffering from rheumatoid arthritis.

## Figures and Tables

**Table 1:** The physicochemical characterization of LNPs.

		LNP-si*TNF-α*	LNP-si*TNF-α*-HCQ
Size (nm)		15 ± 3	160 ± 3
PDI		0.29 ± 0.02	0.33 ± 0.003
ζ-potential (mV)		−0.7 ± 1.0	−1.0 ± 0.7
EE%	siTNF-α	89.8 ± 1.3	89.2 ± 2.1
	HCQ	/	16.4 ± 1.8

EE: encapsulation efficiency.

## Data Availability

The original contributions presented in this study are included in the article. Further inquiries can be directed to the corresponding authors.
